# Diminishing returns: Nudging Covid-19 prevention among Colombian young adults

**DOI:** 10.1371/journal.pone.0279179

**Published:** 2022-12-22

**Authors:** Allen Blackman, Bridget Hoffmann

**Affiliations:** 1 Climate and Sustainable Development Sector, Inter-American Development Bank, Washington, DC, United States of America; 2 Research Department, Inter-American Development Bank, Washington, DC, United States of America; Universidade Regional do Noroeste do Estado do Rio Grande do Sul, BRAZIL

## Abstract

Nonpharmaceutical interventions (NPIs) like social distancing, face masks, and handwashing will continue to be a frontline defense against Covid-19 for some time. But their effectiveness depends critically on compliance by young adults, who are most likely both to become infected and to infect others. We conducted a randomized controlled trial in Bogotá, Colombia, to assess the effectiveness of informational nudges emphasizing the private and public benefits of compliance on university students’ concern about Covid-19, recent compliance with NPI recommendations, and intended future compliance. Although nudges boosted concern, they had limited effects on either recent or intended future compliance. We attribute these null results to high baseline levels of information about and compliance with NPIs, an informational diminishing returns scenario that is likely to be increasingly common globally.

## 1. Introduction

Nonpharmaceutical interventions (NPIs) like social distancing, face masks, and handwashing have been a cornerstone of the global policy response to the Covid-19 pandemic. Given persistent vaccine hesitancy, shortages of vaccines and other pharmaceutical interventions, and the emergence of new variants that are resistant to these interventions, NPIs are likely to continue to be a frontline defense for some time.

But their effectiveness depends critically on compliance by young adults. The reason is that young adults are most likely to become infected and therefore infect others [1]. For example, people aged 20 to 29 accounted for 19 percent of all reported cases of Covid-19 worldwide in 2020, the largest percentage of all 10-year age cohorts, even though they only comprised 15 percent of the world’s population [2]. Numerous studies have documented how young adults, who are often asymptomatic [3], can be Covid-19 “superspreaders.” For example, Oster et al. [4] examined county-level case data in the United States and found that surges in the general population were typically preceded by smaller surges in infections among persons less than 25 years old. Furuse et al. [5] and Laxminarayan et al. [6] reach similar conclusions using data for Japan and India. Among young adults, university students are a particular concern [7–9].

Unfortunately, an incentive problem complicates efforts to boost young adults’ compliance with NPI recommendations. For people of all ages, noncompliance entails a negative externality: individuals who choose not to comply are not only more likely to become infected but also more likely to infect others, and therefore they do not pay the full social cost of their choice. However, this incentive problem is particularly severe for young adults, since older people, not younger ones, are at highest risk of serious illness and death from Covid-19 [10, 11]. For example, in the United States, even though people aged 18 to 29 have represented more than a fifth of confirmed Covid-19 cases, they have accounted for less than 1 percent of deaths [2]. Perhaps because of this incentive problem, compliance with Covid-19 NPI recommendations tends to be relatively low among young adults [12–14].

In developing countries, the urgency of boosting young adults’ compliance is heightened by two factors. Young adults generally make up a much larger share of the population than in industrialized countries [15]. In addition, multigenerational households and extensive intergenerational contact speed the spread of infection from young adults to older, more vulnerable people [11, 16].

Informational nudges have been widely recommended to encourage compliance with NPI recommendations [17–20] and have been employed in both industrialized and developing countries [21, 22]. From a policy perspective, it is important to understand whether such nudges can improve young adults’ compliance, and also what types of nudges are likely to be most effective. A primary consideration is whether the messages should emphasize the private benefits of compliance to the recipient (she is less likely to get infected and seriously ill) or the public benefits to others (she is less likely to infect others, who may become seriously ill). The relative effectiveness of private versus public benefit NPI framing has been studied in a variety of public health contexts, including hand washing, vaccination, and second-hand smoke [23–26]. A priori, each type of message could be expected to have an impact on young adults’ Covid-19 NPI compliance, and empirical evidence is needed to determine which is more effective.

We conducted a randomized controlled trial (RCT) involving 1,221 university students in Bogotá, Colombia, to assess the effectiveness of three informational treatments—one emphasizing the private benefits of compliance, a second emphasizing the public benefits, and a third emphasizing both public and private benefits—on concern about Covid-19, recent compliance with five NPI recommendations, and intended future compliance. Our study contributes to the emerging experimental evidence on the effects of informational interventions on compliance with Covid-19 NPIs. To our knowledge, none of this evidence focuses on young adults and little on developing countries. Results are decidedly mixed. As for the randomized controlled trials (RCTs) in developing countries, Banerjee et al. [27] and Boruchowicz et al. [28] find that text messages in West Bengal, India and São Paulo, Brazil speeded NPI adoption. Although Banerjee et al. [27] do not find statistically significant differences in effects of message variants emphasizing the public and private benefits of compliance, Boruchowicz et al. [28] conclude that those emphasizing public benefits were most effective. On the other hand, however, Bahety et al. [29] are not able to discern an effect of any of a range of different text message variants on knowledge about or adoption of NPIs in Bihar, India.

As for industrialized country RCTs, on one hand, Carfora and Catelani [30], Jordan et al. [31], Lunn et al. [32], Sasaki et al. [33], and Utych and Fowler [34] find positive effects of informational nudges on attitudes, recent compliance, and/or intended future compliance in Italy, the United States, Ireland, Japan and the United States respectively. On the other hand, however, Barari et al. [14], Favero and Pedersen [35], Hacquin et al. [36] and Sanders et al. [37], are not able to discern an effect of nudges on attitudes and beliefs about and/or intended compliance with NPIs in Italy, the United States, France, and the United Kingdom respectively. Finally, working in Denmark and the United Kingdom, Falco and Zaccagni [38] and Hume et al. [39] also are unable to discern effects on recent compliance but do find positive effects on intended future compliance.

We make three contributions to this experimental literature on the effects of informational nudges on compliance with Covid-19 NPIs. First, as noted above, to our knowledge, our study is the first to analyze the behavior of young adults and one of a small number to focus on a developing country. Young adults’ compliance will be critical to combating Covid-19 in developing countries. Second, whereas most Covid-19 nudge experiments rely on cross-sectional data collected using anonymous web survey services, we collected panel data, administered our treatments and surveys in relatively small, proctored web conferencing sessions, and complemented our main treatments with an interactive email campaign—features designed to enable us to study both intended future compliance and recent compliance, to reduce inattention, and to ensure adherence to study protocols. And finally, to our knowledge, ours is the first study to show that informational nudges emphasizing the private benefits of compliance boost participants’ concern about Covid-19’s effects on their friends and communities, a finding that suggests such messages can effectively do double duty.

## 2. Context

Colombia’s population, which totals just over 50 million, is younger than that of most industrialized countries. Forty-nine percent of Colombians are younger than 30, with 18 percent in their 20s [15]. By contrast, only 39 percent of the US population is younger than 30, with 14 percent in their 20s.

**Fig 1:**
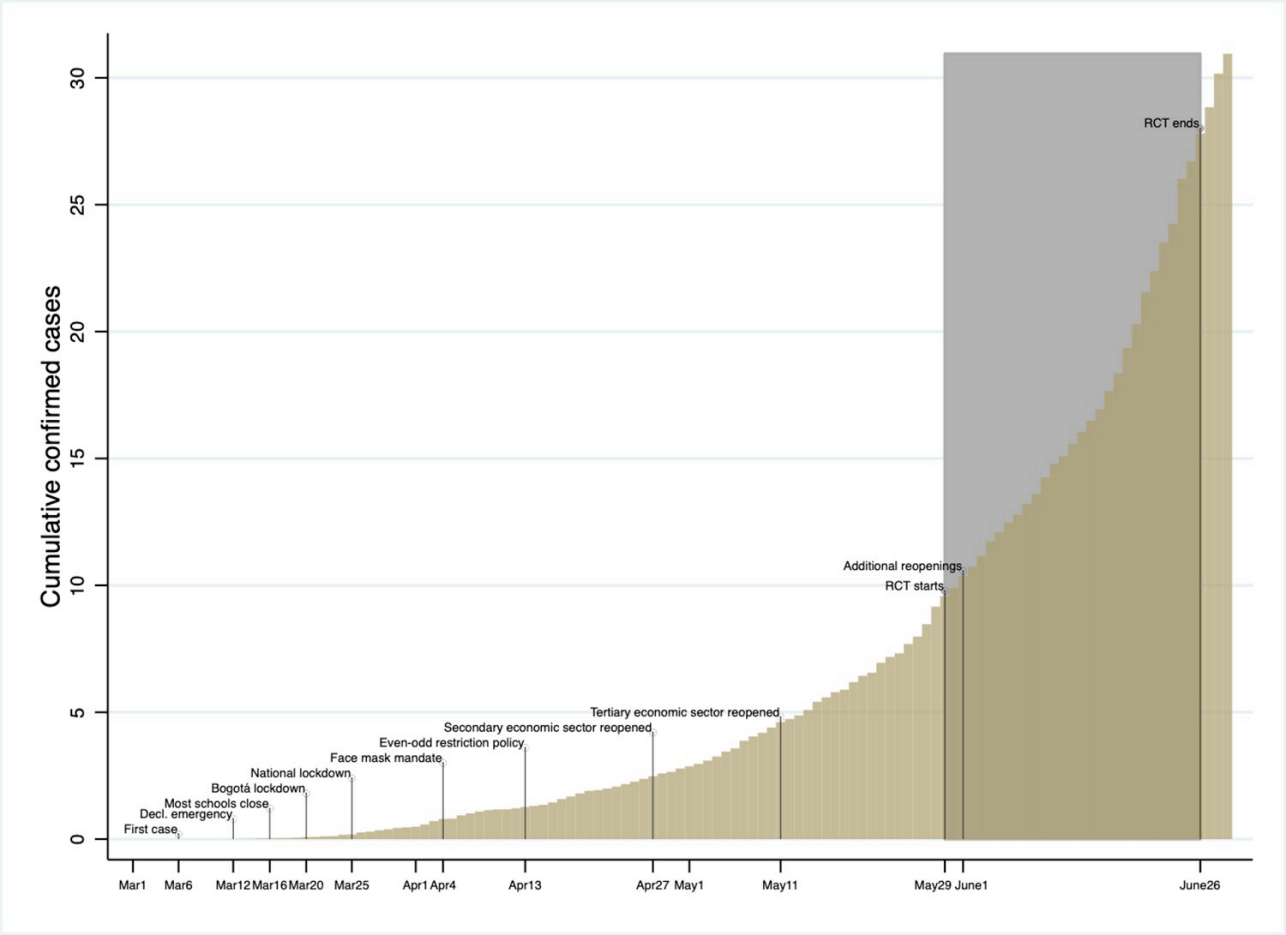
Timing of experiment.

The first case of Covid-19 in Colombia was reported in Bogotá on March 6, 2020 (Fig 1). By May 25, four days before our experiment began, the country counted 21,981 cases and 750 deaths, and Bogotá counted 7,386 cases and 212 deaths [40]. As in other countries, young adults accounted for a relatively large share of cases and small share of deaths. As of May 25, 22 percent of the cases in the country were among people in their 20s, whereas 15 percent were among people 60 and older [40]. However, only 2 percent of deaths were among people in their 20s whereas 73 percent were among people 60 or older. These percentages were similar for Bogotá.

City, national, and private sector leaders instituted a variety of policy responses during the two and a half months before our experiment began, including a state-of emergency declaration (March 12), a citywide lockdown (March 20), and a countrywide stay-at-home order (March 25) [41, 42] (Fig 1). These measures were followed by a gradual sector-by-sector lifting of restrictions during the late Spring and Summer, starting with those sectors considered most vital.

Throughout these months, national and city authorities used television, radio, and social media to promote a range of NPIs, including the five on which our RCT focuses—washing hands, wearing a face mask in public, cleaning frequently touched surfaces, staying home whenever possible, and social distancing [43–46]. Universities in Bogotá implemented their own NPI protocols and information campaigns [47–52].

## 3. Experimental design

We used a 2×2 factorial design to assess the effectiveness of three informational treatments on young adults’ attitudes and behaviors.

### 3.1. Treatments

All three treatments provided very similar contextual information and health recommendations; for the most part, they differed only in how they motivated the recommendations (for the full text of our treatment materials, see **S1 Text**). The common contextual information included the following:

- neither a vaccine nor a targeted treatment exists;
- Covid-19 is 30 times more infectious than the common flu;
- as of May 25, 21,981 Covid-19 cases and 750 deaths had been reported in Colombia and 7,386 cases and 212 deaths had been reported in Bogotá;
- people most at risk of serious illness are those over 60 years of age and with certain comorbidities: asthma, cardiovascular conditions, diabetes, kidney disease, and hepatitis;
- young adults have the highest risk of infection; and
- the risk of serious illness to young adults is not insignificant.

In addition, all treatments recommended five NPIs:

- *hand washing*: frequently, using soap, and for at least 20 seconds immediately after being in a public place, touching a frequently touched surface, coughing, or sneezing;
- *face mask*: covering the mouth and nose, worn at all times in public;
- *cleaning*: frequently touched household surfaces at least once per day;
- *stay home*: except for buying food, obtaining medical care, or other activities critical for survival; and
- *social distancing*: avoid proximity to others closer than 2 meters in public places.

The motivation for complying with the five NPI recommendations differed across the three treatments (for these summary statements, we use virtually the same wording as Jordan et al. [31]):

- *private*. Emphasis on private benefits: “Coronavirus is a serious threat to you. You must take this threat very seriously to avoid contracting Covid-19 and becoming gravely ill or dying. Fortunately, there are five steps you can take to keep yourself safe.”
- *public*. Emphasis on public benefits: “Coronavirus is a serious threat to your community. You must take this threat very seriously to avoid spreading Covid-19 to vulnerable groups and causing them to die. Fortunately, there are five steps you can take to prevent the spread of Covid-19 in vulnerable groups in your community.”
- *combined*. Emphasis on both private and public benefits: “Coronavirus is a serious threat to you and your community. You must take this threat very seriously to avoid contracting Covid-19 and becoming gravely ill or dying or spreading Covid-19 to vulnerable groups, causing them to die. Fortunately, there are five steps you can take to keep yourself and your community safe.”

As discussed below, participants were randomly assigned either to one of the three treatment groups or to a pure control group.

### 3.2. Outcomes

In our baseline and endline surveys, we collected information about three sets of outcomes, each consisting of five outcomes (Table 1). The first set aims to capture concerns about Covid-19. Using a four-point Likert scale, with one being the lowest level and four the highest, respondents indicated the following: *likelihood of infection*, their self-assessed likelihood of contracting Covid-19; *concern self*, their level of concern about getting seriously ill from Covid-19; *concern friends*, their level of concern about infecting friends who then become seriously ill; *concern household*, their level of concern about infecting members of their household who then become seriously ill; and finally, *concern community*, their level of concern about infecting members of their community other than family and friends who then become seriously ill.

**Table 1:** Variables and means at baseline.

Variable	Units	Definition	Nobs.	Mean
Treatments				
*private*	0/1	emphasizes benefits of mitigation for respondent	318	0.26
*public*	0/1	emphasizes benefits of mitigation for vulnerable groups	327	0.27
*combined*	0/1	emphasizes benefits of mitigation for respondent and vulnerable groups	346	0.28
*control*	0/1	placebo treatment on classical music	230	0.19
Concern				
*likelihood infection*	[1–4]	likelihood respondent will get infected with Covid19	1214	2.67
*concern self*	[1–4]	if infected, concern respondent will have serious health effects	1219	2.67
*concern friends*	[1–4]	if infected, concern friends will have serious health effects	1218	3.34
*concern household*	[1–4]	if infected, concern household will have serious health effects	1208	3.70
*concern community*	[1–4]	if infected, concern community member will have serious health effects	1219	3.18
*concern index*	n/a	index of 5 concern outcomes	1221	0.00
Recent compliance				
*hand washing*	%	% of times over past 7 days washed hands when should have	1195	76.80
*face mask*	%	% of times over past 7 days wore a mask while outside	1219	93.57
*cleaning*	days	days over past 7 that cleaned frequently touched surfaces	1163	3.90
*stay home*	days	days over past 7 that stayed home except for critical trips	1209	5.98
*social distancing*	%	% of times over past 7 days maintained 2 meters’ distance	1210	80.26
*recent compliance index*	n/a	index of 5 recent compliance outcomes	1221	0.02
Intended compliance				
*hand washing intention*	%	% of times over next 7 days intend to wash hands when should	1211	90.52
*face mask intention*	%	% of times over next 7 days intend to wear a mask while outside	1212	94.81
*cleaning intention*	days	days over next 7 that intend to clean frequently touched surfaces	1165	5.07
*stay home intention*	days	days over past 7 that intend to stay home except for critical trips	1173	6.17
*social dist*. *intention*	%	% of times over next 7 days intend to maintain 2 meters’ distance	1209	88.53
*intended compliance index*	n/a	index of 5 intended compliance outcomes	1220	0.00
Characteristics				
*older*	0/1	≥ 22 years old	1221	0.32
*female*	0/1	female	1219	0.57
*poor*	0/1	*estrato*^a^ < = 2	1215	0.32
*work*	0/1	work outside home	1215	0.06
*relatives in hh*	0/1	live with parents and/or other relatives	1221	0.90
*no*. *people in hh*	no.	no. people in household	1216	3.01
*elder in hh*	0/1	cohabitate with someone 60 years or older	1200	0.31
*elder parent*	0/1	have parent 60 years or older	1221	0.21
*health*	0/1	respondent’s overall health is very bad to moderate (≤ 4 of 7)	1221	0.26
*comorbidity self*	0/1	respondent has Covid19 comorbidity	1218	0.10
*comorbidity parents*	0/1	respondent knows parent has Covid19 comorbidity	1220	0.32
*left wing*	0/1	respondent’s political ideology is left-wing	1221	0.33
*right wing*	0/1	respondent’s political ideology is right-wing	1221	0.11
*knows Covid19 case*	0/1	respondent personally knows someone diagnosed with Covid19	1221	0.15
*knows Covid19 death*	0/1	respondent personally knows someone who died from Covid19	1221	0.03
*localidad*	0/1	administrative unit within Bogotá (19 binary dummies)^b^	--	--

^a^*Estratos* are socioeconomic categories used by Colombian municipal governments to charge differential fees and taxes for public services and to allocate various benefits. The six *estratos* are 1 (low-low), 2 (low), 3 (medium-low), 4 (medium), 5 (medium-high), and 6 (high).

^b^The 19 administrative units (*localidades*) are Antonio Nariño, Barrios Unidos, Bosa, Chapinero, Ciudad Bolívar, Engativá, Fontibón, Kennedy, La Candelaria, Los Mártires, Puente Aranda, Rafael Uribe, San Cristóbal, Santa Fé, Suba, Teusaquillo, Tunjuelito, Usaquén, and Usme.

The second set of outcomes comprises self-reported rates of compliance with the five NPI recommendations during the seven days preceding the survey. For *hand washing*, *face mask*, and *social distancing*, respondents reported the percentage of all the times over the past seven days when they should have followed this recommendation that they actually did so. For *cleaning* and *stay home*, respondents reported the number of days out of the last seven that they followed this recommendation. As discussed in Section 3.3, at least seven days elapsed between the baseline session, during which baseline survey data were collected and then the initial information sessions were administered, and the endline sessions during which endline survey data were collected. A complementary email informational treatment was administered during this interim period between the baseline and endline sessions. Hence, the estimating equations detailed in Section 5 effectively test whether our three treatments caused participants to change their compliance with the five NPI recommendations in a week-long period after receiving an initial informational treatment, during which they received a complementary email treatment.

The third set of outcomes comprises intentions to comply with each of the five NPI recommendations during the seven days following the survey. For *hand washing intention*, *face mask intention*, and *social distancing intention*, respondents reported the percentage of the times over the next seven days when they should follow this recommendation that they intend to do so. For *cleaning intention* and *stay home intention*, respondents reported the number of the next seven days that they intend to follow this recommendation.

While the exact wording of our outcome measures and the survey questions that underpin them, were not taken directly from other studies, they are similar to measures used in the literature. For example, our recent compliance measures are similar to those used in Bahety et al. [29], Banerjee et al. [27], Boruchowicz et al. [28], Falco and Zaccagni [38], Hume et al. [39], and Saski et al. [33]; our intended compliance outcome measures are similar to those used in Carfora and Catelani [30], Favero and Pedersen [35], Hacquin et al. [36], Hume et al. [39], Jordan et al. [31], Lunn et al. [32], Sasaki et al. [33], and Utych and Fowler [34]; and our concern measures are similar to those used in Banerjee et al. [27] and Favero and Pedersen [35].

In addition to the three sets of five outcomes described above, we generate a participant-level summary index for each set: *concern index*, *recent compliance index*, and *intended compliance index*. Following Kling et al. [53], each index is an equally weighted average of the z-scores of all five individual outcomes, oriented such that a positive sign indicates greater concern or compliance. The z-scores, in turn, are created by subtracting the mean of the outcome in the control group and dividing by the standard deviation of the outcome in the control group, so that z-scores have a mean of 0 and a standard deviation of 1 in the control group.

The indices are helpful for three reasons. The first has to do with exposition: indices are a convenient means of summarizing overall concern about Covid-19, overall recent compliance with all five NPI recommendations, and overall intended compliance. But the indices have importance beyond exposition. A common theme in the literature on NPIs is that because all NPIs have limitations, any single NPI is insufficient to slow the spread of an infectious disease such as Covid-19. Rather, a range of simultaneous NPIs is needed [54–56], an approach often referred to as the Swiss cheese model (because multiple slices laid on top of each other are needed to patch holes in any individual slice). Hence, to the extent this model is correct, our indices can be interpreted as a measure of NPIs’ likely overall effect. Finally, the indices improve statistical power to detect effects that go in the same direction [53].

### 3.3. Sample and logistics

Using both social media and print advertisements, we recruited a sample of 1,349 students 18 years of age or older who were studying at more than 40 universities in Bogotá. As noted above, young adults play a critical role in the spread of Covid-19 and among young adults, those at universities are a particular concern. Participants engaged in three activities: (i) a baseline survey session immediately followed by an information session containing a either a treatment or a placebo presentation, (ii) a one-week interactive email campaign reiterating the treatment or placebo messages, and (iii) an endline survey session.

Because of Covid-19 social distancing requirements, the survey and information sessions were conducted online using a web conferencing platform (Zoom). To verify the identity of participants, maximize their engagement, and ensure compliance with study protocols, these remote sessions were carefully controlled. Students who accepted an invitation to participate in the study were scheduled for a baseline survey and information session and, later, an online endline survey session at a certain date. Participation in each session was capped at 35 students (attendance in all sessions averaged 24.9 participants). All sessions were proctored by at least two members of the research team who checked identification to verify that participants were the university students who had been invited; obtained consent; introduced, explained, and monitored engagement with the surveys; answered procedural questions; and played a PowerPoint presentation providing the informational treatments (prerecorded to ensure consistency across information sessions).

Participants were randomly assigned to treatment and control groups at the baseline survey/information session-level. Randomization was designed to assign 19 percent of the sample to the control group and 27 percent to each of the three treatment groups. As discussed below, actual assignment percentages differed slightly because randomization was not at the individual-level.

Administered using SurveyCTO online software, the baseline and endline surveys, which were scheduled one week apart, elicited information on recent compliance with five NPI recommendations, intended future compliance, attitudes and beliefs about COVID-19 and the NPI measures, and sociodemographic characteristics. Table 1 lists variables derived from the survey data.

As noted above, informational treatments were administered just after the baseline survey. Following that survey, participants could opt to participate in a one-week interactive email campaign intended to reinforce the informational treatment they received. They received three email messages, one following the baseline information session on the same day as that session, and two more over the next seven days. Each provided a brief summary of the information session, highlighting the *public*, *private*, or *combined* motivational information and framing (for the text of a sample email, see **S1 Text**). In addition, to encourage engagement with this summary, the second and third emails offered participants in each treatment group an opportunity to answer a simple question about its content. For example, participants in the *private* treatment group had an opportunity to answer the question, “In the United States, what percentage of young adults who have contracted Covid-19 required hospitalization?”

Study participants were compensated: they received COP 10,000 (US $2.80) for completing the baseline survey and information session, COP 60,000 (US $17.24) for completing the endline survey, and COP 6,000 (US $1.85) for each email question they answered correctly. Payments were made using money transfer smartphone applications.

To minimize attrition in the control group and to ensure that all participants had the opportunity to earn the same compensation, participants in the control group received a placebo treatment (about classical music), were sent three follow-up emails, and had an opportunity to answer comprehension questions.

Study participants were recruited in May 2020. Fifty-four remote baseline survey/information sessions with a total of 1,349 participants were conducted between May 29 and June 26 (Fig 1). Fifty-three remote endline survey sessions with a total of 1,319 participants were conducted between June 5 and 26. After data cleaning, our sample comprises 1,221 participants, implying an overall attrition rate of 7.4 percent. Attrition is balanced across treatments (S1 Table in **S1 Text**). The control group comprised 230 participants (19 percent), the *private* group 318 participants, (26 percent), the *public* group 327 participants (27 percent), and the *combined* group 346 participants (28 percent) (Table 1).

### 3.4. Ethical approval and registration

Written consent was obtained from study participants. Ethical approval for our study was provided by Innovations for Poverty Action’s Institutional Review Board (Protocol No. 15615). The experiment was registered in the American Economic Association Randomized Controlled Trial Registry (AEARCTR-0005876).

## 4. Data

Because participants were randomly assigned to treatments at the baseline information session-level, ex ante we would not expect these assignments to be correlated with potentially confounding participant characteristics. Nevertheless, it is useful to check for residual correlations with the observable participant characteristics on which we collected data at baseline: *older*, *female*, *poor*, *work*, *relatives in hh*, *no*. *people in hh*, *elder in hh*, *elder parent*, *health*, *comorbidity self*, *comorbidity parents*, *left wing*, *right wing*, *knows Covid19 case*, *knows* C*ovid19 death*, and *localidad* (Table 1). As expected, participant characteristic covariates are not jointly significant predictors of the treatments (S2 Table in **S1 Text**). Moreover, only three covariates are correlated with treatments (*comorbidity parents* is correlated with *private* and *public*; *health* is correlated with *combined*; and *work* is weakly correlated with *private*). To control for residual correlations, we include all of the aforementioned participant characteristic covariates as explanatory variables (see Eq 1 below).

Summary statistics highlight three potentially pertinent characteristics of our study sample (Table 1). First, the large majority of participants—90 percent—live with relatives. Second, participants are more concerned about the health effects of Covid-19 for others—particularly family and household members—than for themselves. On average, participants rated both their likelihood of infection and their concern about becoming seriously ill at 2.7 on a four-point Likert scale and their concern about household members at 3.7, friends at 3.3, and community members at 3.2.

**Fig 2:**
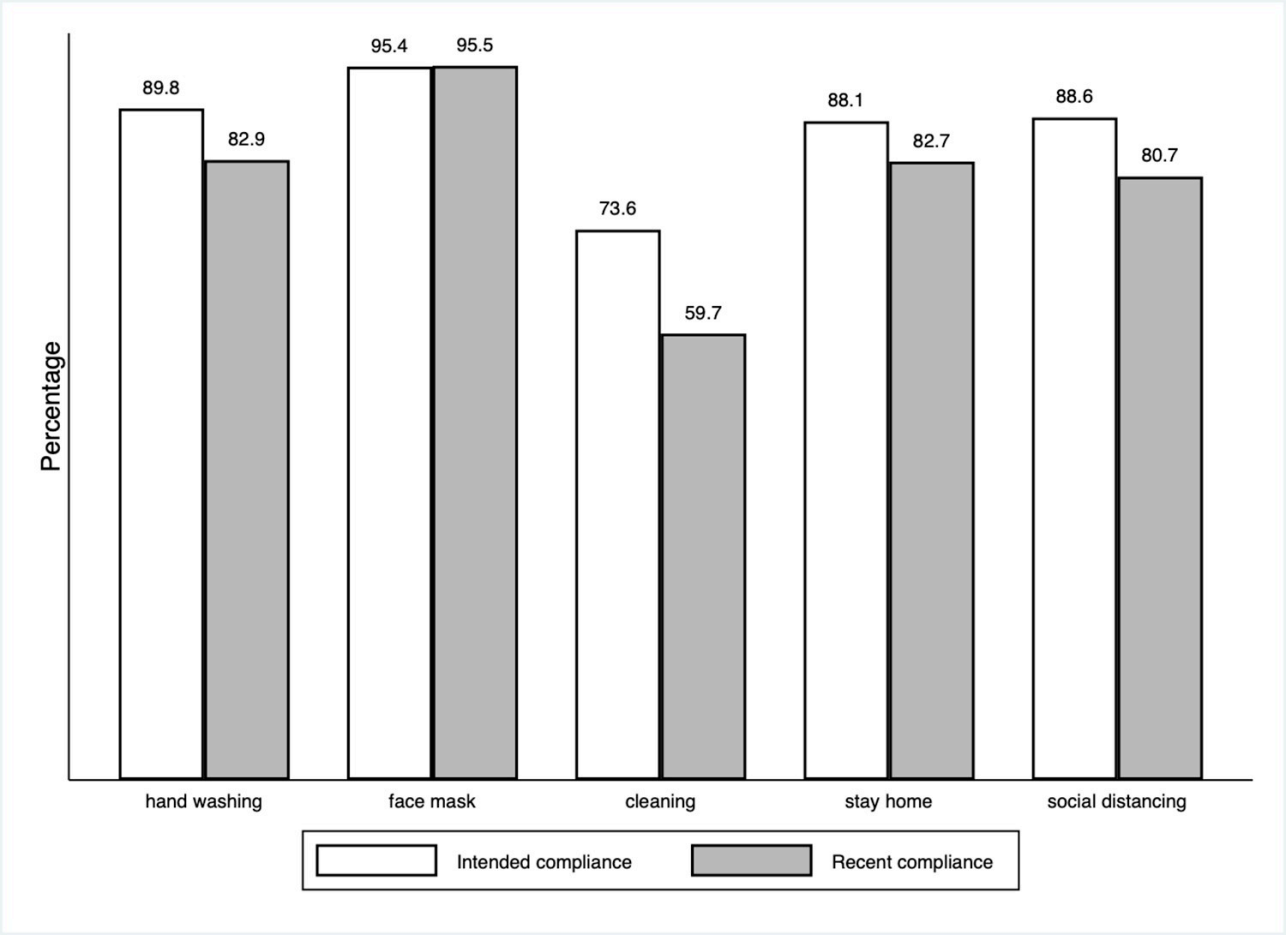
Intention to comply versus recent compliance at baseline for control group^a^. ^a^For this figure, *cleaning* and *stay home*, which are measured in days per week, are converted to percentages by dividing by 7 and multiplying by 100.

And third, baseline levels of compliance with all NPI recommendations are high but, for most NPIs, substantially below rates of intended compliance. Participants reported that in the 7 days before the baseline survey, they washed their hands 77 percent of the times recommended, maintained at least a 2-meter distance in public 80 percent of the times recommended, wore a face mask 94 percent of the times recommended, cleaned frequently touched surfaces 3.9 days of the 7 days recommended, and stayed home 6 of the 7 days recommended. However, for most NPI recommendations, these compliance rates were well below rates respondents said they intended to achieve in the 7 days after the baseline survey (Table 1 and Fig 2). At baseline, participants reported that they intended to wash their hands 91 percent of the times recommended, a 14 percentage point increase, and intended to socially distance 89 percent of the time, a 9 percentage point increase. In addition, they reported that they intended to clean surfaces in their homes 5 days a week, a 2-day increase. Only for the two recommended NPIs mandated by law—wearing a face mask and staying at home—did recent compliance more or less match intentions. S3 Table in **S1 Text** reports on participants’ responses to questions about the reasons for complying and not complying with each NPI recommendation.

Finally, average levels of some outcomes changed between the baseline and endline, among both control and treatment groups. Except for staying at home, recent compliance with all NPI recommendations increased for all control and treatment groups. Fewer rates of intended compliance or levels of concern increased (S4 Table in **S1 Text**).

## 5. Estimating equations

Because our treatments are randomly assigned, estimating their effect on changes between baseline and endline levels of outcomes is straightforward [57]. We use ordinary least squares (OLS) to fit regressions of the form

$$Y=\beta_{1}private+\beta_{2}public+\beta_{3}combined+\beta_{4}y+\beta_{3}x\prime +\epsilon$$ (1)

where *Y* is the outcome at endline; *private*, *public* and *combined* are binary indicator variables for the three treatments; y is the outcome at baseline; *x* is a vector of covariates; *β*_*m*_ is a parameter or vector of parameters; and *ϵ* is an error term. The elements of *x* are *older*, *female*, *poor*, *work*, *relatives in hh*, *no*. *people in hh*, *elder in hh*, *elder parent*, *health*, *comorbidity self*, *comorbidity parents*, *left wing*, *right wing*, *knows Covid19 case*, *knows* C*ovid19 death*, and 18 administrative unit (*localidad*) fixed effects (Table 1). We cluster standard errors at the baseline survey session-level.

To evaluate treatment effect heterogeneity, we use ordinary least squares to fit regressions of the form

$$Y=\beta_{1}private+\beta_{2}public+\beta_{3}combo+{\beta_{4}private\times x_{j}+\beta_{5}public\times x_{j}+\beta_{6}combo\times x_{j}+\beta }_{7}y+\beta_{8}x\prime +\epsilon \;(j=1,2\ldots n)$$ (2)

where *x*_*j*_ is the *j*^th^ element of *x*. As discussed below, to simplify the treatment effect heterogeneity analysis, we focus on a single outcome: *compliance index*. Here, too, we cluster standard errors at the baseline survey session-level.

## 6. Results

In general, we find that our treatments boost concern about Covid-19 infection but have limited overall effects on both recent compliance and intended compliance.

### 6.1. Concern

**Fig 3:**
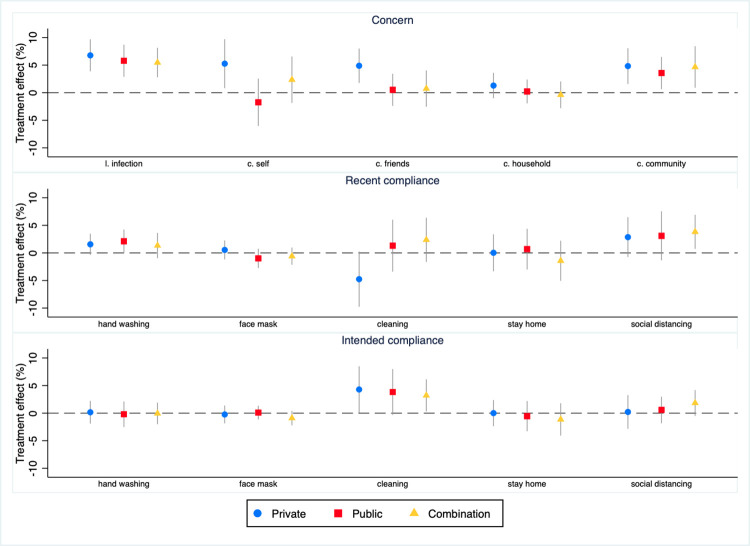
Estimated treatment effects^a^. ^a^For this figure, treatment effects for *cleaning* and *stay home*, which are measured in days per week, are converted to percentages by dividing by 7 and multiplying by 100; l.p. = Likert scale points; p.p. = percentage points; circles, squares and diamonds are point estimates and whiskers are 90 percent confidence intervals.

Our treatments had significant effects on four of our five Covid-19 concern outcomes: all except *concern household* (Table 2 and Fig 3). All three treatments boost *likelihood infection*. The magnitude of these effects, all of which are highly significant, is similar across treatments, ranging from 0.17 to 0.20 Likert scale points, equivalent to a 7–8 percent increase above counterfactual levels. The *private* treatment increases three concern outcomes: it boosts *concern self* by 0.13 Likert scale points, *concern friends* by 0.17 Likert scale points, and *concern community* by 0.17 Likert scale points. All these effects are equivalent to a 5 percent increase above the counterfactual level. Only the *private* treatment has a significant (positive) effect on the *concern index*.

**Table 2:** Treatment effects; ordinary least squares regression results and minimum detectable effects (MDEs).

Panel A: Concern outcomes						
	*likelihood infection*	*concern self*	*concern friends*	*concern household*	*concern community*	*concern index*
*private*	0.203***	0.133**	0.174**	0.019	0.174**	0.159***
	(0.047)	(0.066)	(0.070)	(0.048)	(0.070)	(0.045)
MDE	0.131	0.186	0.196	0.135	0.196	0.127
MDE/Counterfact. (%)^a^	5.199	7.198	6.098	3.641	6.098	--
*Public*	0.173***	-0.042	-0.029	-0.029	-0.029	0.039
	(0.049)	(0.074)	(0.064)	(0.044)	(0.064)	(0.043)
MDE	0.137	0.208	0.179	0.123	0.179	0.122
MDE/Counterfact. (%)^a^	5.466	8.064	5.555	3.302	5.555	
*Combined*	0.171***	0.075	-0.022	-0.045	-0.022	0.072
	(0.042)	(0.068)	(0.069)	(0.053)	(0.069)	(0.045)
MDE	0.117	0.189	0.193	0.147	0.193	0.127
MDE/Counterfact. (%)^a^	4.651	7.333	6.019	3.963	6.019	--
Observations	1074	1077	1076	1062	1076	1079
R-squared	0.386	0.408	0.169	0.073	0.169	0.229
Counterfactual	2.512***	2.578***	3.215***	3.714***	3.215***	0.022***
	(0.035)	(0.056)	(0.049)	(0.031)	(0.049)	(0.035)
Panel B: Recent compliance outcomes						
	***hand washing***	***face mask***	***Cleaning***	***stay home***	***social distancing***	***recent comp*. *index***
*Private*	0.884	0.299	-0.272**	0.007	0.513	0.002
	(0.926)	(0.891)	(0.128)	(0.129)	(1.683)	(0.033)
MDE	2.594	2.496	0.358	0.362	4.714	0.093
MDE/Counterfact. (%)^a^	3.059	2.595	8.529	6.238	5.687	--
*Public*	1.661*	-1.142	-0.033	0.063	1.565	0.027
	(0.980)	(1.095)	(0.137)	(0.126)	(2.046)	(0.039)
MDE	2.744	3.067	0.383	0.352	5.728	0.108
MDE/Counterfact. (%)^a^	3.236	3.189	9.137	6.078	6.910	--
*Combined*	0.852	-0.996	0.088	-0.097	2.185	-0.000
	(1.066)	(0.962)	(0.108)	(0.142)	(1.523)	(0.036)
MDE	2.984	2.695	0.301	0.397	4.263	0.101
MDE/Counterfact. (%)^a^	3.518	2.801	7.180	6.848	5.143	--
Observations	1058	1073	1014	1063	1063	1079
R-squared	0.384	0.167	0.537	0.187	0.277	0.447
Counterfactual	84.815***	96.194***	4.196***	5.797***	82.889***	0.038***
	(0.775)	(0.683)	(0.072)	(0.115)	(1.380)	(0.025)
Panel C: Intended compliance outcomes						
	***hand washing intention***	***face mask intention***	***cleaning intention***	***stay home intention***	***social dist*. *intention***	***intended comp*. *index***
*Private*	-0.069	-0.305	0.216*	-0.047	0.394	0.013
	(1.142)	(0.885)	(0.113)	(0.090)	(1.686)	(0.050)
MDE	3.198	2.479	0.317	0.252	4.721	0.139
MDE/Counterfact. (%)^a^	3.442	2.541	6.509	4.116	5.273	--
*Public*	-0.374	-0.451	0.208	-0.051	-0.102	0.004
	(1.233)	(0.724)	(0.126)	(0.106)	(1.416)	(0.042)
MDE	3.453	2.027	0.353	0.296	3.965	0.116
MDE/Counterfact. (%)^a^	3.717	2.078	7.238	4.823	4.429	--
*Combined*	-0.504	-1.218	0.210***	-0.083	1.807	0.007
	(1.088)	(0.841)	(0.070)	(0.110)	(1.323)	(0.040)
MDE	3.047	2.354	0.195	0.309	3.703	0.112
MDE/Counterfact. (%)^a^	3.279	2.414	3.995	5.039	4.136	--
Observations	1066	1068	999	1011	1066	1078
R-squared	0.331	0.099	0.588	0.217	0.281	0.438
Counterfactual	92.920***	97.529***	4.876***	6.130***	89.529***	0.022***
	(0.989)	(0.441)	(0.055)	(0.077)	(1.209)	(0.033)

The dependent variable is the endline concern or compliance level. Independent variables are *private*, *public*, *combined*, the baseline compliance or concern level, and the following covariates: *older*, *female*, *poor*, *work*, *relatives in hh*, *no*. *people in hh*, *elder in hh*, *elder parent*, *poor health*, *comorbidity self*, *comorbidity parents*, *left wing*, *right wing*, *knows Covid19 case*, *knows* C*ovid19 death*, and (n = 18) *localidad* fixed effects. Standard errors are clustered at baseline survey session-level. The counterfactual is the average rate of compliance predicted by estimated regression equation with all treatment dummy variables equal to zero.

^a^Not calculated for index because index components are standardized to have mean zero in the control group and as a result, the counterfactual is close to zero.

*** p<0.01

** p<0.05

* p<0.1.

As for the relative efficacy of the three treatments, it is notable that the *private* treatment has a statistically significant effect on four of the five outcomes—all except *concern household*—whereas the *public* and *combined* treatments have statistically significant effects on only one, *likelihood infection*. In addition, as just noted, only the *private* treatment has a statistically significant effect on the *concern index*. In the case of the single outcome where more than one treatment has a statistically significant effect—*likelihood infection*—we are not able to reject the null hypothesis that all three treatment effects are equal.

### 6.2. Recent compliance

Although seven of the estimated effects of our treatments on the concern outcomes are statistically significant, only two of the estimated effects on recent compliance outcomes are. For *hand washing*, the *public* treatment boosts percentage compliance by 1.7 percentage points, a 2 percent increase above the counterfactual rate (Table 2 and Fig 3). However, this effect is only weakly significant. For *cleaning*, the *private* treatment reduces percentage compliance by 0.27 days, a 7 percent decrease below the counterfactual. None of the three treatments are statistically significant in the *recent compliance index* regression.

To determine whether these null results are due to a lack of statistical power, we calculate minimum detectible effects (MDEs) (Table 2). A MDE is the smallest true absolute value of the treatment effect that has at least an X percent chance of producing a statistically significant estimate, given the size and variability of the study sample (i.e., the smallest true absolute value of the treatment effect for which there is less than a 1–X percent chance of making a Type II error; [58]). It can be calculated as a simple multiple of the estimated standard error of the treatment effect. Following convention [59], we use X equal to 80 percent. In addition, we allow for a two-sided hypothesis test and a 5 percent significance level (equivalently, a one-sided test and a 2.5 percent significance level). Given these assumptions, the MDE is 2.8 times the standard error. For the five individual NPI outcomes, MDEs range from 2.6 to 9.1 percent of the counterfactual compliance rate or level, and they average 5.3 percent. The implication is that our models have the power to identify changes in compliance larger than 2.6–9.1 percent above or below counterfactual levels 80 percent of the time.

### 6.3. Intended compliance

Only two of the estimated intended behavior treatment effects are statistically significant, both for the *cleaning intention* outcome. The *private* treatment boosts intended compliance by 0.22 days, a 4.4 percent increase above the counterfactual (Table 2 and Fig 3). However, this effect is only weakly significant. The combined treatment increases intended compliance by 0.21 days, a 4.2 percent increase above the counterfactual. None of the three treatments are statistically significant in the *intended compliance index* regression.

For the five individual intention-to-comply outcomes, MDEs range from 2.1 to 7.2 percent of the counterfactual compliance rate or level and average 4.2 percent (Table 2). Hence, our models have the power to identify changes in compliance larger than 2.1–7.2 percent above or below counterfactual levels 80 percent of the time.

### 6.4. Treatment effect heterogeneity

The finding that our informational nudges have limited effects on the NPI recent compliance for all participants in our sample begs the question of whether these nudges might have significant effects on certain subgroups. If they did, then policymakers could target nudges to these subgroups. As discussed in Section 5, to address that question, we rely on interaction terms (Eq 2). We use the *recent compliance index* as our sole outcome variable, for two reasons. First, as discussed above, a common theme in the literature is that to effectively stem Covid-19’s spread, what matters most is overall compliance across a range of NPIs, not compliance with any particular NPI. In addition, using a single outcome simplifies the analysis and makes results easier to interpret.

**Fig 4:**
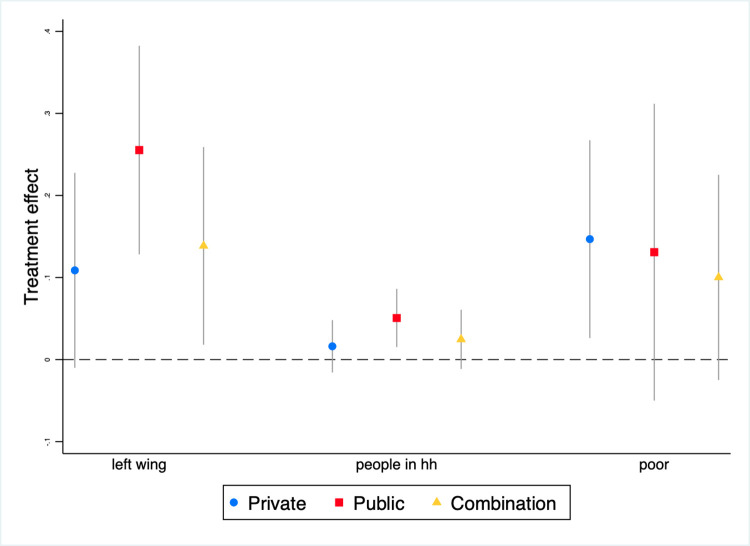
Treatment effect heterogeneity^a^. ^a^Circles, squares and diamonds are point estimates and whiskers are 90 percent confidence intervals.

Regression results suggest that our nudges improved overall NPI compliance among subgroups comprising participants who identified as politically left-wing, lived with a relatively large number of people, and were relatively poor (Table 3 and Fig 4). For left-wing participants, both the *public* treatment and the *combined* treatment improved overall compliance. For participants living with more people, the *public* treatment raised compliance. And for participants living in relatively poor households, the *private* treatment boosted compliance. Recall that these results are generated with three regressions each with a single interaction term. However, regression results are quite similar when all three interaction terms are included in the same regression (S5 Table in S1 Text).

**Table 3:** Treatment effect heterogeneity for recent compliance index; ordinary least squares regression results.

Treatments	Interaction covariate		
	*left wing*	*no*. *people in hh*	*poor*
*private*	-0.040	-0.046	-0.045
	(0.045)	(0.066)	(0.030)
*public*	-0.060	-0.128	-0.019
	(0.050)	(0.079)	(0.057)
*combined*	-0.052	-0.074	-0.035
	(0.045)	(0.064)	(0.032)
*private×covariate*	0.109	0.016	0.147**
	(0.071)	(0.019)	(0.072)
*public×covariate*	0.255***	0.051**	0.131
	(0.076)	(0.021)	(0.108)
*combined×covariate*	0.139*	0.025	0.100
	(0.072)	(0.022)	(0.075)
Observations	1079	1079	1079
R-squared	0.452	0.450	0.449

Each of the three columns on the right represents a distinct regression. In each, the dependent variable is the endline *recent compliance index*. Independent variables are the baseline *recent compliance*, *index*, *older*, *female*, *poor*, *work*, *relatives in hh*, *no*. *people in hh*, *elder in hh*, *elder parent*, *poor health*, *comorbidity self*, *comorbidity parents*, *left wing*, *right wing*, *knows Covid19 case*, *knows* C*ovid19 death*, and (n = 18) *localidad* fixed effects. Standard errors are clustered at baseline survey session-level.

*** p<0.01

** p<0.05

* p<0.1.

## 7. Discussion

### 7.1. Effects on concerns versus behaviors

Why did our nudges affect concern but not recent compliance or intended future compliance? First, it is important to emphasize that for the most part, our nudges did not affect either of these sets of outcomes. The implication is that the reason nudges failed to boost recent compliance does *not* have to do with a gap between intentions and behaviors, which is frequently blamed for the failure of nudges to have the intended impacts [60, 61]. That is, it is not the case that our nudges motivated participants to want to boost their compliance, but that for whatever reason—forgetfulness, competing priorities, a tendency to overstate intentions—they did not follow through. Rather, for the most part, our nudges did not even cause participants to ratchet up their intentions.

We hypothesize that our null effects on recent and intended compliance stem from two factors, both related to the fact that our nudges were administered roughly two months after the start of the pandemic in Bogotá. First, by that time, our participants were saturated with information about Covid-19 and with NPI recommendations, and as a result, even though our nudges may have provided some new information, they probably did not dramatically affect participants’ basic understanding of the pandemic or NPIs. As noted in Section 2, in the two months preceding our experiment, national and local authorities were actively disseminating information about Covid-19 and all five NPI recommendations on which our study focuses, and they also mandated compliance with two: *face mask* and *stay home*. Students were even more exposed to this information than the average Bogotá resident because, as discussed in Section 2, they were directly affected by mandated school closures and because their universities promulgated their own Covid-19 protocols and information campaigns. In addition, they had near universal access to the internet.

Second, as a result of this information saturation, our participants’ baseline levels of compliance with NPI recommendations were fairly high. For the three NPI recommendations where compliance was measured in percentages, baseline levels ranged from 77 to 94 percent, and for the two recommendations measured in days per week, it ranged from 3.9 to 6.0 days. Marginal costs of compliance are undoubtedly increasing in the level of compliance. As a result, even though we do not observe marginal compliance costs, at baseline, they were likely relatively high. In other words, ceiling effects blunted our nudges’ impact.

We hypothesize that, notwithstanding these two barriers to changes in recent and intended compliance—information saturation and high marginal compliance costs—our nudges affected most of our concern outcomes because the marginal costs of changing concerns are lower than those of changing behavior. Our nudges likely provided at least some new information about the pandemic, such as statistics on its effects on young adults and the risks to vulnerable groups, and even if they did not, they provided a salient reminder about the seriousness of the disease.

Our null results comport with findings of other experiments testing the effect of informational nudges on compliance with Covid-19 NPI recommendations albeit using study samples of adults of all ages (Section 1). Perhaps most relevant is Bahety et al. [29], one of the few such RCTs conducted in a developing country, which finds that a range of different text message variants, including those emphasizing the private versus public benefits, had no discernable effect on knowledge about or adoption of NPIs in Bihar, India. They attribute this result at least partly to the fact that by the time their study was fielded 5 to 6 months after the start of the pandemic, their target population was already well-informed about NPIs and marginal costs of compliance had risen. Perhaps less surprising is that NPI nudge experiments focused on industrialized countries, where one might expect higher baseline levels of knowledge and compliance, reach similar conclusions [14, 35–39].

### 7.2. Private versus public motivations

Because most of our estimated treatment effects for recent and intended compliance are insignificant, our ability to generate inferences about the relative efficacy of nudges emphasizing private versus public benefits of compliance with NPI recommendations is limited. As discussed above, we do find some evidence that the *private* treatment is more effective in boosting concern about Covid-19 than the *public* or *combined* treatments: the *private* treatment has a statistically significant effect on four of the five concern outcomes, whereas the *public* and *combined* treatments have statistically significant effects on only two.

Beyond that, it is notable that the *private* treatment boosted participants’ concern about Covid-19’s effects on their friends and community. We hypothesize that this finding reflects the fact that participants’ friends and to a lesser extent members of their broader community are likely to be young adults. Therefore, the *private* treatment—which emphasizes risk to young adults—conveys a message that friends and community members also are at risk. In other words, the self-oriented message here turns out to have an other-oriented effect. To our knowledge, this finding is new to the literature.

### 7.3. Treatment effect heterogeneity

We find that our treatments were more effective at boosting recent compliance among subgroups of participants who identified as politically left-wing, lived with more people, and were relatively poor (Section 6.4). How do these findings compare with those from similar RCTs? To our knowledge, only few RCTs examine heterogeneous treatment effects for similar subgroups. Several of their findings align with ours. Focusing on the United States, Jordan et al. [31] find that nudges are more effective among politically liberal participants and Favero and Pederson [35] find that intended compliance with social distancing is higher among Democrats. Working in Brazil, Boruchowicz et al. [28] find that nudges are more effective in keeping relatively poor people from leaving home (for certain types of trips, namely exercising and dog walking).

What causal mechanisms might explain our subgroup effects? Our data do not enable us to definitively identify mechanisms, and therefore our discussion is necessarily speculative. That said, we hypothesize that nudges were more effective among participants who were left-wing and who lived with more people because at baseline, these participants were predisposed to view protecting vulnerable groups and *not* protecting oneself as an important benefit of NPI compliance. As a result, these participants were more likely to find the nudges emphasizing benefits of NPIs for vulnerable groups to be persuasive. Two elements of our results and survey data support that hypothesis. First, only the two treatments that emphasized the benefits of NPI compliance for vulnerable groups (*public* and *combined*) had discernible effects in these subgroups; the treatment that emphasized benefits for young adults (*private*) did not (Table 3 and Fig 4). Second, our survey data on stated reasons for compliance indicate that participants in these subgroups were more likely to choose “want to avoid infecting family” or “want to avoid infecting cohabitators” as most important reason for complying (Tables 2 and 4).

**Table 4:** Mechanisms for subgroup effects: Participant characteristics, by subgroup.

Subgroup	Characteristic	
	*protect vulnerable groups compliance motive* ^a^	*concern self*
*left wing* = 0	0.62	2.71
*left wing* = 1	0.68	2.58
t-test^b^	**	*
*large hh*^c^ = 0	0.62	2.65
*large hh* = 1	0.68	2.72
t-test^b^	**	
*poor* = 0	0.63	2.60
*poor* = 1	0.66	2.82
t-test^b^		***

^a^Indicator variable = 1 if selected “want to avoid infecting family” or “want to avoid infecting cohabitators” as most important reason for complying for at least 3 of 5 nonpharmaceutical interventions.

^b^Test of null hypothesis that means are not equal.

^c^Binary indicator if household size exceeds median (3 persons).

*** p<0.01

** p<0.05

* p<0.1.

We conjecture that nudges were more effective among participants who were poor because the health and livelihood costs they expected to incur if infected with Covid-19 were relatively high. As a result, they were more likely to find nudges emphasizing benefits of NPIs for young adults to be persuasive. This hypothesis is supported by the fact that only the treatment emphasizing the benefits of NPI compliance for young adults (*private*) increased compliance for this subgroup; the treatments emphasizing benefits to vulnerable groups (*public* and *combined*) did not. In addition, poor participants had higher average baseline levels of concern that if they were infected, Covid-19 would have serious health consequences for themselves (Table 4). Finally, research confirms that in Bogotá, the poor can in fact expect to incur greater health and livelihood costs if infected with Covid-19. Poor households mainly rely on public health clinics, not private doctors and hospitals, and as a result do not have access to health care on par with richer households [62]. And in general, poor households suffer disproportionate economic effects from Covid-19, in part because they lack the resources to mitigate economic shocks [63].

### 7.4. Strengths, limitations, and external validity

Our study has both strengths and limitations. As for strengths, as noted in Section 1, it fills a gap in the evidence base on Covid-19 NPIs. To our knowledge, it offers the first experimental evidence on the compliance with Covid-19 NPI recommendation of young adults in a developing country, a group whose compliance has been critical to combating the global pandemic. In addition, as mentioned in Sections 1 and 3, it incorporates design elements aimed at enhancing the reliability of our data and analysis. It features panel (versus cross-sectional) data, treatments and surveys administered in relatively small, proctored (versus in anonymous unsupervised) web conferencing sessions, and a treatment comprised of both a live information session and an interactive email campaign (versus simpler remote information provision).

As for limitations, given budget and logistical constraints, we rely on a convenience sample of university students rather than a random sample of young adults. As discussed below, this sampling strategy has implications for external validity. In addition, our data on NPI compliance are self-reported. In principle, self-reported data on recent compliance could be biased upward if respondents tend to provide answers that conform to perceived social norms [64, 65]. This bias could in turn affect our results if it were correlated with our treatments—that is, if nudges to comply with NPIs create additional incentives for participants to overreport compliance. However, at least two factors provide reassurance. First, our broad qualitative finding is that our nudges did not boost recent compliance. Therefore, self-report bias would explain our results only if it caused participants to underreport compliance, which seems quite unlikely. Second, emerging empirical research on Covid-19 NPI compliance suggests that self-report bias is small. For example, three recent studies each using a different method to detect deviations between actual and self-reported compliance with Covid-19 NPI recommendations (list experiments, cross-wise models, and analysis of smartphone location data) all conclude that these deviations are quite small or negligible [66–68].

To what extent are our findings externally valid? A number of factors suggest they might not generalize to other countries. First, the participants in our study sample are not likely to be representative of the average developing country young adult in terms of income, education, exposure to NPI recommendations, and a variety of other potentially confounding factors. Among developing countries, Colombia is relatively well off—it is classified by the World Bank as an upper middle country, the second highest of that institution’s four gross national income per capita categories. Moreover, the participants in our experiment were university students in Colombia’s capital city. As a result, even compared to the average Colombian young adult, they likely enjoyed above-average socioeconomic status, educational attainment, and access to health care. And finally, Bogotá’s policy response to the Covid-19 pandemic (see Section 2) was likely more robust than that in many developing country cities.

Notwithstanding these factors, as discussed above, our results largely comport with existing experimental evidence on the efficacy of informational nudges in boosting compliance with NPI recommendations, In particular, they jibe with the findings of Bahety et al. [29], one of the few such RCTs conducted in a developing country, which finds that a range of different text message variants had little effect on knowledge about or adoption of NPIs in Bihar, India. In the final analysis, additional studies of young adult compliance with Covid-19 NPI recommendations are needed to determine whether our findings generalize to other countries.

### 7.5. Policy implications

Keeping in mind the uncertainty noted above about external validity, our findings have at least four implications for policymakers. First, although others have highlighted the inherent challenges of trying to boost compliance with NPI recommendations among young adults [12–14], our study indicates that some young adult characteristics may facilitate such efforts: they tend to live with their relatives and to be concerned about infecting them and others. Second, the timing of nudges likely moderates their effectiveness: during a pandemic, when information about the disease and NPI recommendations is plentiful, efficacy likely attenuates over time as recipients become saturated with information, as their compliance rates increase, and as the marginal costs of compliance rise. Third, even when nudges to young adults have limited efficacy, they may still be effective among subpopulations with certain observable characteristics—those who are politically left-wing, living with more people, and relatively poor. As a result, policymakers may be able to enhance the efficacy of nudges by targeting them to identifiable subgroups. Our treatment could be scaled up at relatively low cost by, for example, disseminating a pre-recorded treatment video like the one we used via the internet, television and radio and by automating an email campaign. And finally, among young adults, nudges emphasizing the private benefits of compliance may effectively do double duty, heightening concern about the entire target group.

## 8. Conclusion

We conducted a RCT in Bogotá, Colombia to assess the effectiveness of three informational treatments—one emphasizing the private benefits of compliance, one the public benefits, and one both types of benefits—on university students’ concern about Covid-19, recent compliance with NPI recommendations, and intended future compliance. We draw three main conclusions. First, although our nudges boosted participants’ concern about Covid-19, they had limited effects on both recent compliance with the five NPI recommendations and intended future compliance. We hypothesize that these null results stem from the fact that our nudges were administered more than two months after the start of the pandemic in Colombia, by which time participants had already been exposed to considerable information about NPIs and had already ratcheted up compliance—an informational diminishing returns scenario likely to be increasingly common globally. Second, the treatment emphasizing the private benefits of compliance to young adults not only increased participants’ concern about the effect of Covid-19 on them but also heightened their concern about their friends and communities, a result we attribute to the fact that participants’ friends, and to a lesser extent members of their broader community, are likely to be young adults. And third, our treatments were more effective at boosting recent compliance among certain subgroups—namely, participants who identified as politically left-wing, lived with more people, and were relatively poor.

## Supporting information

S1 Text Supporting information. (DOCX)
