# Supplementary material for: Diminishing returns: Nudging Covid-19 prevention among Colombian young adults
Source: PLoS One. 2022 Dec 22;17(12):e0279179. doi: 10.1371/journal.pone.0279179 (PMC9778522; doi:10.1371/journal.pone.0279179)
Supplement: S1 Text — (DOCX) [file pone.0279179.s001.docx]

**Supporting Information**

**Diminishing Returns: Nudging Covid-19 Prevention**

**Among Colombian Young Adults**

Short title: Nudging Covid-19 Prevention

Allen Blackman

(corresponding author)

Inter-American Development Bank

1300 New York Ave. NW

Washington, DC 20577

v: +1202-714-8034 f: +1859-425-3363

[allenb@iadb.org](mailto:allenb@iadb.org)

Bridget Hoffmann

Inter-American Development Bank

[bridgeth@iadb.org](mailto:bridgeth@iadb.org)

**CONTENTS**

1. Reasons for Complying and not Complying with Nonpharmaceutical Interventions
2. Treatment and Control Group PowerPoints and Emails

References

Tables

Figures

**1. REASONS FOR COMPLYING AND NOT COMPLYING WITH NONPHARMACEUTICAL INTERVENTIONS**

In addition to asking about past and intended compliance with NPI recommendations, our surveys asked participants to report the most important reason for complying with each recommendation and the most important reason for not complying. Echoing out finding that participants tended to be more concerned about others than themselves, for all five NPI recommendations, the majority of respondents, ranging from 56 to 72 percent, reported that the most important reason for complying at baseline was to avoid infecting their family (Table S3). The second-largest share of respondents, 21 to 32 percent, said the most important reason was to avoid becoming seriously ill.

[Insert Table S3 here]

As for noncompliance, for hand washing, wearing face masks, and cleaning, the largest share of participants, ranging from 49 to 81 percent, reported that the most important reason was forgetting to comply. Notably, however, for wearing face masks, almost a third of respondents said the most important reason was that they were uncomfortable. For staying home, participants attributed noncompliance to a variety of activities, including shopping (21 percent), sports and exercise (18 percent), taking a walk (18 percent), and socializing (13 percent). Finally, the plurality (43 percent) said the most important reason for not complying with the social distancing recommendation was that it was difficult in crowded locations.

**2. TREATMENT AND CONTROL GROUP POWERPOINTS AND EMAILS**

**2.1. General notes**

PowerPoints were presented in the information sessions immediately after the baseline survey. This file contains the English translation of the text of that PowerPoint. The verbal presentation that accompanied it closely followed the text and was prerecorded to ensure consistency.

Each study participant received three emails, one just after the baseline information session, on the same day as that session, and two more over the next seven days. This file contains the English translation of the text of the first email. The subsequent two emails were identical except for (i) the introductory language in the second paragraph, describing the progress of the email campaign, and (ii) the questions at the end of the email. The first email did not contain a question. The second and third emails each contained a different question. Both questions are included below.

**2.2. Private treatment**

2.2.1. PowerPoint text

Slide 1: What is Covid-19?

- An infectious disease that causes respiratory conditions
- 30 times more deadly than the common flu
- Scientists have not developed a vaccine or treatment

Slide 2: Covid-19 is extremely contagious

- The majority of those infected do not exhibit symptoms for weeks
- From Wuhan, China, it has spread to 190 countries around the world in four months

Slide 3. Covid-19 in Colombia

- Infections and deaths have increased exponentially since March

|  | *Colombia*  *(as of May 25)* | *Bogotá*  *(as of May 25)* | *Colombia predictions*  *(December 31)* |
| --- | --- | --- | --- |
| *Confirmed cases* | 21,981 | 7,386 | 4,000,000 |
| *Deaths* | 750 | 212 | 3,000-80,000 |

Slide 4. People at highest risk of serious illness or death

- Older than 60 years
- Individuals with underlying health conditions such as asthma, cardiovascular disease, diabetes, kidney and liver disease

Slide 5. People at highest risk of infection: Young adults

- In the case of Korea

|  | *Percentage of total population* | *Percentage of cases of Covid-19* |
| --- | --- | --- |
| *People between 60-69 years old* | 12 | 12 |
| *People between 20-29 years old* | 13 | 30 |

Slide 6. Young adults can also become seriously ill or die

- In the case of the United States, for persons 20–44 years old with Covid-19
  - 21 percent were hospitalized
  - 4 percent were referred to intensive care
  - 0.2 percent died

Slide 7. Covid-19 is a serious risk to you!

- You must take this threat very seriously to avoid contracting Covid-19 and becoming seriously ill or dying
- Fortunately, there are five things you can do to avoid getting seriously ill or dying

Slide 8. Wash your hands

- Frequently
- Using soap and water
- For at least 20 seconds
- Especially immediately after
  - Being in a public place
  - Touching an object or a surface frequently touched by other people
  - Coughing, sneezing, blowing your nose

Slide 9. Use a face mask or cloth covering

- Cover your mouth and nose
- Always use in places outside your home

Slide 10. Clean and disinfect surfaces that you touch frequently

- Every day

Slide 11. Stay in your house

- Stay in your house except to buy food, visit a doctor, or do other activities that are critical for survival

Slide 12. Avoid close contact with other people

- Maintain a distance of 2 meters from people outside your home at all times

2.2.2. Email

Thank you for participating in the study organized by Innovations for Poverty Action-IPA and Rosario Experimental and Behavioral Economics Lab-REBEL for the Inter-American Development Bank-IDB.

This email provides key information on the coronavirus disease (Covid-19) from the online session in which you participated today. In the next week, you will receive two similar emails along with an invitation to answer a question about this information. A correct response will increase by COP 6000 the COP 60,000 compensation you will be paid if you agree to participate in a follow-up online survey.

Key message: Covid-19 is a serious threat to you. You should take this threat very seriously to avoid contracting Covid-19 and getting seriously ill or dying.

The reasons are that Covid-19:

- Is 30 times more deadly than the ordinary flu, is extremely contagious, and has caused 212 deaths in Bogotá through May 25, 2020;
- Is much more likely to be contracted by young adults than older adults; for example, in Korea, people in their 20s are twice as likely to be infected as people in their 60s;
- Creates severe health risks for young adults; for example, in the United States, among people aged 20–44 who contracted Covid-19, 21% required hospitalization, 4% required intensive care, and 0.2% died.

Therefore,

- It is important to prevent young adults from getting infected to prevent them from becoming seriously ill or dying.

Fortunately, there are five steps you can take to keep yourself safe:


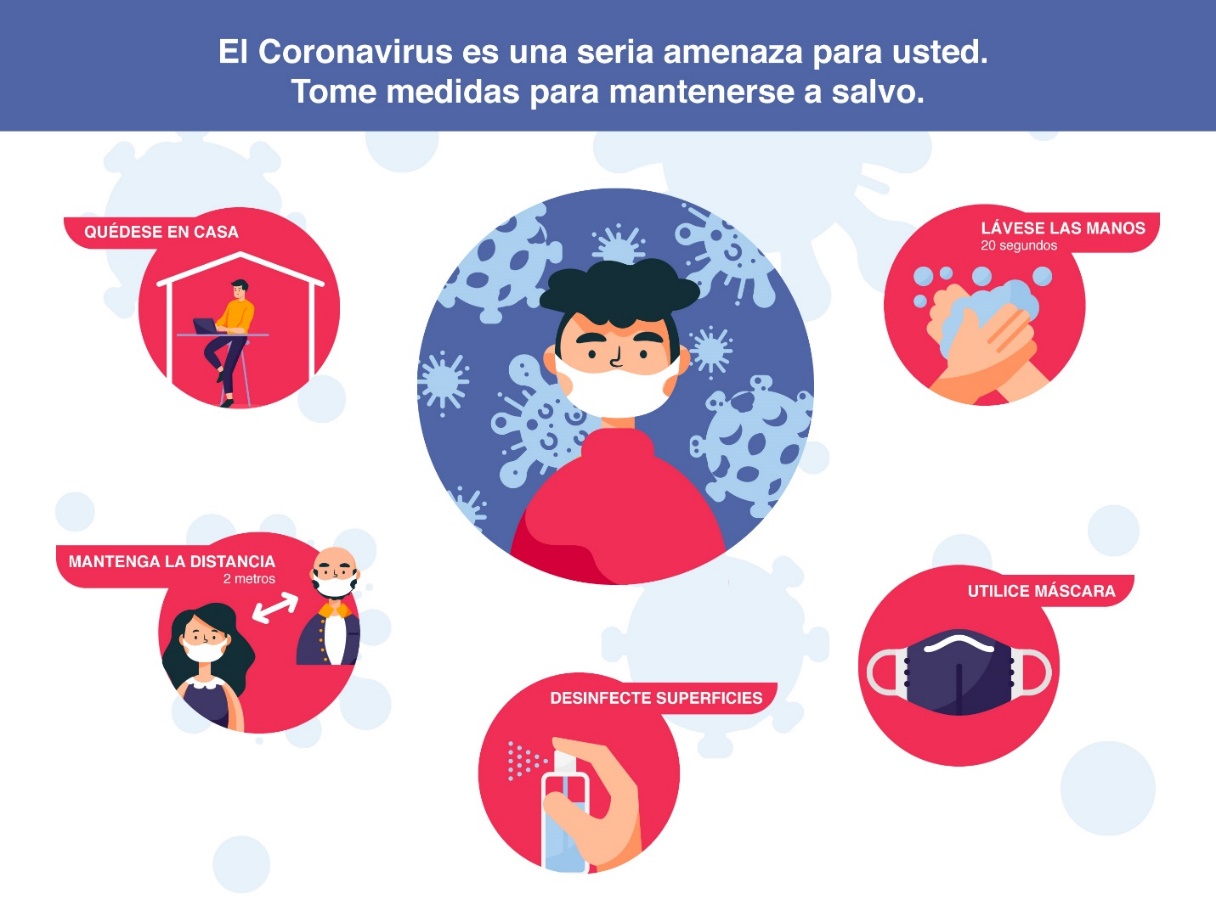


Question (included in the second email, not the third): In the United States, what percentage of young adults who have contracted Covid-19 have required hospitalization?

Question (included in the third email, not the second): In the United States, what percentage of young adults who have contracted Covid19 have required intensive care?

**2.3. Public treatment**

2.3.1. PowerPoint text

Slides 1–5. Same as private treatment

Slide 6. Preventing young adults from becoming infected is critical to limiting deaths from Covid-19

- They have a high probability of becoming infected
- They have a high probability of infecting those at high risk of death
  - People older than 60
  - Individuals with underlying health conditions

Slide 7. Covid-19 is a serious risk to your community!

- You must take this threat very seriously to prevent the spread of Covid-19 in vulnerable groups and causing them to die
- Fortunately, there are five steps you can take to prevent the spread of Covid-19 among vulnerable groups in your community

Slides 8–12. Same as private treatment

2.3.2. Email

Thank you for participating in the study organized by Innovations for Poverty Action-IPA and Rosario Experimental and Behavioral Economics Lab-REBEL for the Inter-American Development Bank-IDB.

This email provides key information on the coronavirus disease (Covid-19) from the online session in which you participated today. In the next week you will receive two similar emails along with an invitation to answer a question about this information. A correct response will increase by COP 6000 the COP 60,000 compensation you will be paid if you agree to participate in a follow-up online survey.

Key message: Covid-19 is a serious threat to your community. You should take this threat very seriously to avoid spreading Covid-19 to vulnerable groups and causing them to get seriously ill or die.

The reasons are that Covid-19:

- Is 30 times more deadly than the ordinary flu, is extremely contagious, and has caused 212 deaths in Bogotá since March until May 25, 2020;
- Is most likely to kill people over 60 and people with underlying health conditions;
- Is far more likely to be contracted by young adults than older adults; for example, in Korea, Covid-19 is twice as likely to be contracted by people aged 20–29 as people aged 60–69.

Therefore,

- It is important to prevent young adults from getting infected to slow the spread of the disease to people at highest risk of getting seriously ill or dying.

Fortunately, there are five steps you can take to keep your community safe.


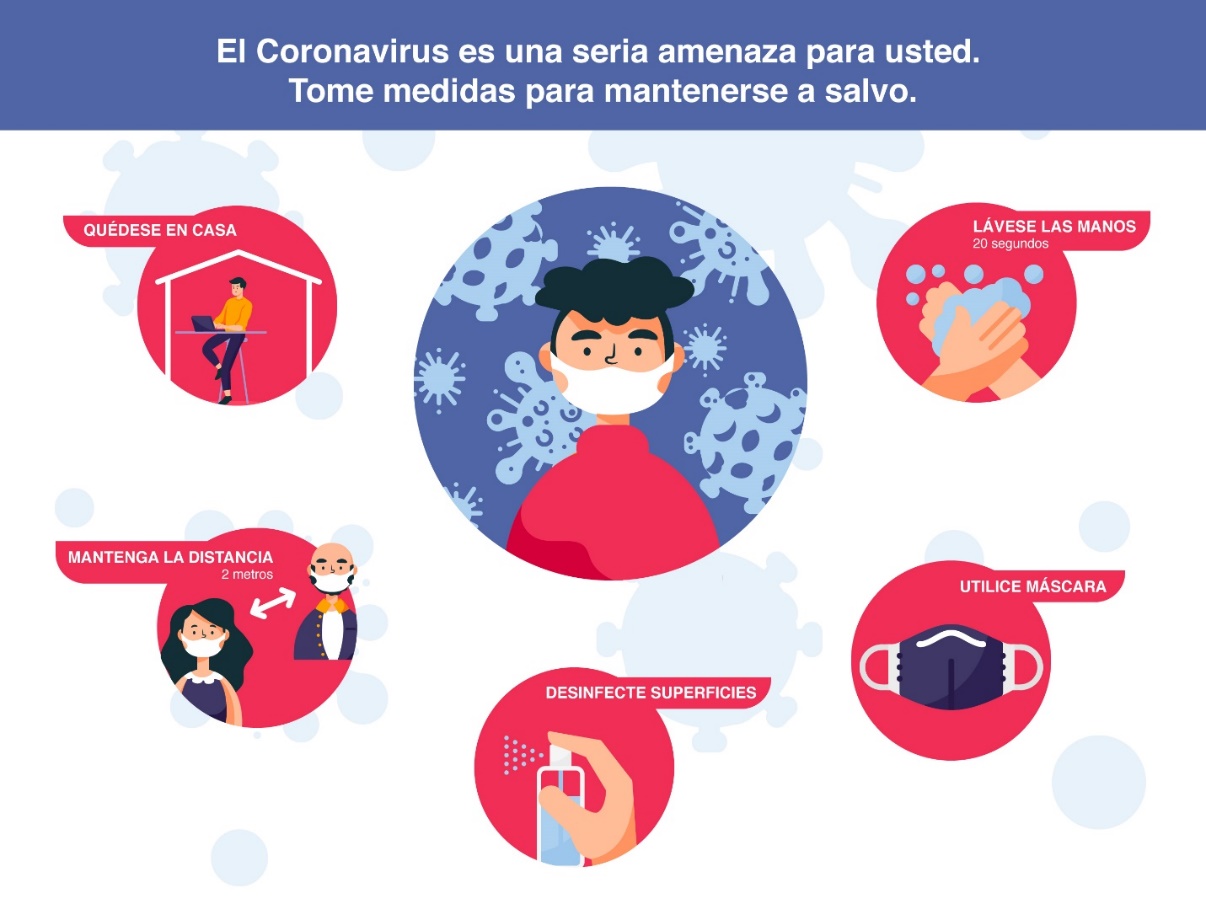


Question (included in the second email, not the third): True or false? Preventing young adults from becoming infected can reduce deaths among people over the age of 60 and with underlying health conditions.

Question (included in the third email, not the second): Which of the following statements is true?

1. Young adults are more likely than older adults to get Covid-19 but are less likely to die from Covid-19.
2. Young adults are less likely than older adults to get Covid-19 and are less likely to die of Covid-19.
3. Young adults are less likely than older adults to get Covid-19 and are more likely to die from Covid-19.

**2.4. Combined treatment**

2.4.1. PowerPoint text

Slides 1–5. Same as private treatment

Slide 6. Same as public treatment slide 6

Slide 7. Same as private treatment slide 6

Slide 8. Covid-19 is a serious risk for you and your community.

- You must take this threat very seriously to prevent contracting Covid-19 and becoming seriously ill or dying or spreading Covid-19 in vulnerable groups, causing them death.
- Fortunately, there are five steps you can take to prevent the spread of Covid-19 among vulnerable groups in your community.

Slides 9–13. Same as private treatment slides 8–12.

2.4.2. Email

Thank you for participating in the study organized by Innovations for Poverty Action-IPA and Rosario Experimental and Behavioral Economics Lab-REBEL for the Inter-American Development Bank-IDB.

This email provides key information on the coronavirus disease (Covid-19) from the online session in which you participated today. In the next week you will receive two similar emails along with an invitation to answer a question about this information. A correct response will increase by COP 6000 the COP 60,000 compensation you will be paid if you agree to participate in a follow-up online survey.

Key message: Covid-19 is a serious threat to both you and your community. You should take this threat very seriously to avoid contracting Covid-19 and getting very ill or dying and to avoid spreading Covid-19 to vulnerable groups and causing them to get seriously ill or die.

The reasons are that Covid-19:

- Is 30 times more deadly than the ordinary flu, is extremely contagious, and has caused 212 deaths in Bogotá since March until May 25, 2020;
- Creates most severe health risks for people over 60 and with underlying health conditions;
- Also creates severe health risks for young adults; for example, in the United States, among people aged 20–44 who contracted Covid-19, 21% required hospitalization, 4% required intensive care, and 0.2% died;
- Is far more likely to be contracted by young adults than older adults; for example, in Korea, Covid-19 is twice as likely to be contracted by people aged 20–29 as people aged 60–69.

Therefore,

- It is important to prevent young adults from getting infected both to prevent them from getting serious ill or dying and to slow the spread of the disease to people at highest risk of getting seriously ill or dying.

Fortunately, there are five steps you can take to keep you and your community safe.


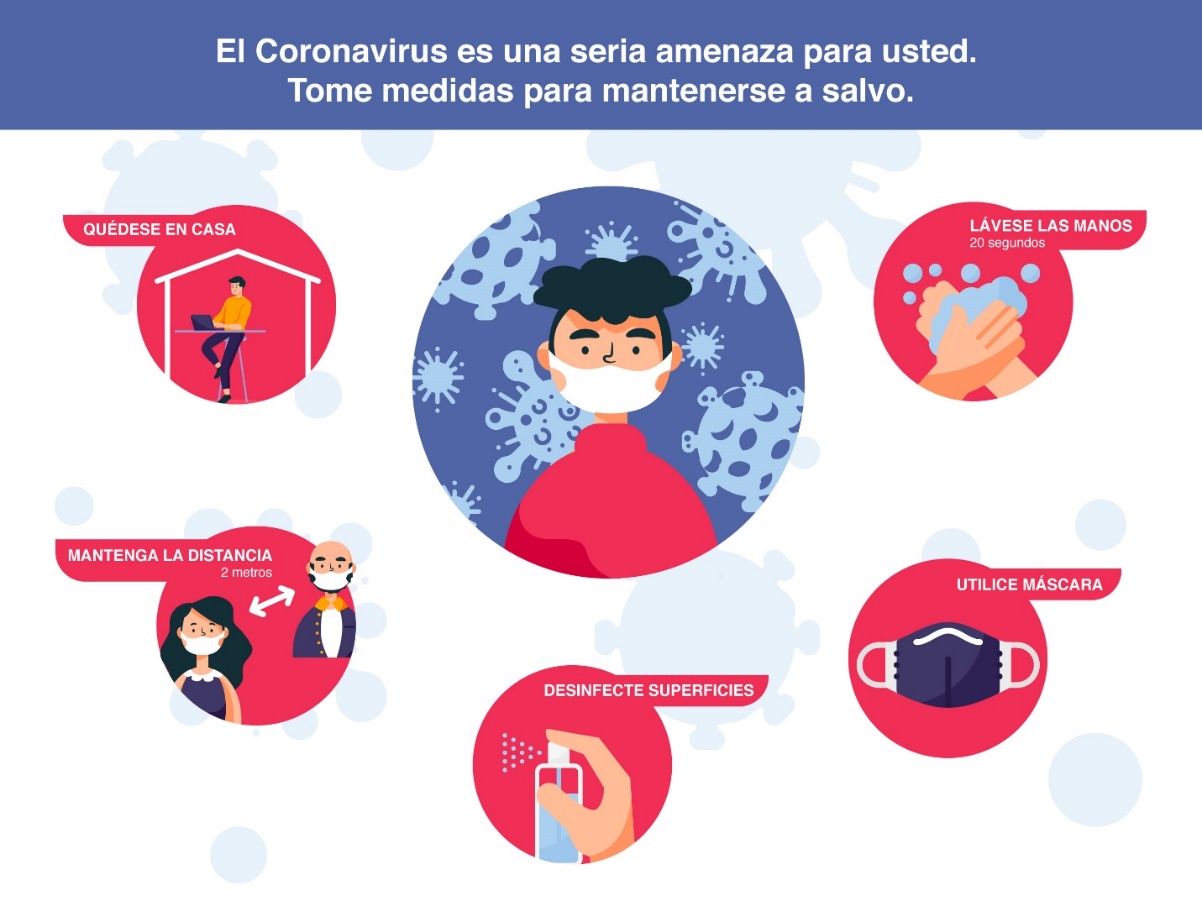


Question (included in the second email, not the third): True or false? Preventing young adults from becoming infected can reduce cases among people over the age of 60 and with underlying health conditions.

Question (included in the third email, not the second): In the United States, what percentage of young adults who have contracted Covid-19 required hospitalization?

**2.5. Control group (placebo treatment)**

2.5.1. PowerPoint text

Slide 1. Welcome! We are going to learn a little about Vivaldi’s Four Seasons

Slide 2. The Four Seasons

- Four concertos for violin published in 1723
- Anthony Vivaldi’s best-known work
- Today more than 1,000 distinct versions exist

Slide 3. The Four Seasons

- Is famous because it is a delight to the ear
- People say that it is a perfect imitation of nature

Slide 4. The Four Seasons

- Did you know that there is a poem behind the music?
- The music is perfectly synchronized to the poem

Slide 5. Principal themes of each season according to the poem

- Spring: the birds say good morning with a happy song
- Summer: Turtledove sings his name "Tortorella" in Italian, before a hailstorm flattens the fields
- Autumn: It brings hunters eager to go out in search of game
- Winter: The storm comes with snow, thunder, and lightning

Slide 6. The Four Seasons

- Only became popular in the 19th century

Slide 7. The Four Seasons

- Vivaldi believed it was important to attract audiences

Slide 8. The Four Seasons

- Vivaldi thought that music was a diversion for everyone

2.5.2. Email

Thank you for participating in the study organized by Innovations for Poverty Action-IPA and Rosario Experimental and Behavioral Economics Lab-REBEL for the Inter-American Development Bank-IDB.

This email provides key information about the concerto 'Four Seasons,' composed by the violinist Antonio Vivaldi, from the online session in which you participated today. In the next week you will receive two similar emails along with an invitation to answer a question about this information. A correct response will increase by COP 6000 the COP 60,000 compensation you will be paid if you agree to participate in a follow-up online survey.

Key message: The Four Seasons concerto teaches us that nature can be represented through musical sounds

The reasons are:

- Experts say this song is a perfect imitation of nature through musical sounds
- Vivaldi spent long hours listening and observing the landscape to translate it into music
- The music is perfectly synchronized with the verses of a poem that describes the landscape and the beings that inhabit it for each season
- The song was published in 1723 but today there are more than 1,000 different versions that have been reproduced in movies and musicals, among others

Therefore,

- From Vivaldi's song, we can affirm that sometimes music represents real scenes and makes us see nature through its sounds


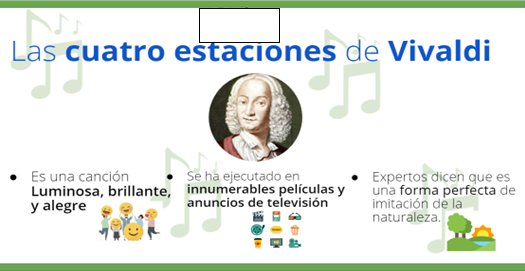


Question (included in the second email, not the third): In what year was the music the Four Seasons released?

Question (included in the third email, not the second): Yes or no: Is Vivaldi’s Four Seasons music synchronized with the verses of a poem?

**REFERENCES**

Allcott, Hunt, Levi Boxell, Jacob Conway, Matthew Gentzkow, Michael Thaler, and David Yang. 2020. Polarization and public health: Partisan differences in social distancing during the coronavirus pandemic. *Journal of Public Economics* 191: 104254.

Bahety, Girija, Sebastian Bauhoff, Dev Patel, and James Potter. 2021. Texts don’t nudge: An adaptive trial to prevent the spread of COVID-19 in India. *Journal of Development Economics* 153: 102747.

Banerjee, Abhijit, Marcella Alsan, Emily Breza, Arun G. Chandrasekhar, Abhijit Chowdhury, Esther Duflo, Paul Goldsmith-Pinkham, and Benjamin Olken. 2020. Messages on COVID-19 prevention in India increased symptoms reporting and adherence to preventive behaviors among 25 million recipients with similar effects on non-recipient members of their communities. Working Paper 27496. Cambridge, MA: National Bureau of Economic Research.

Barari, Soubhik, Stefano Caria, Antonio Davola, Paolo Falco, Thiemo Fetzer, Stefano Fiorin, Lukas Hensel, Andriy Ivchenko, Jon Jachimowicz, Gary King, Gordon Kraft-Todd, Alice Ledda, Mary MacLennan, Lucian Mutoi, Claudio Pagani, Elena Reutskaja, Christopher Roth, and Federico Raimondi Slepoi. 2020. Evaluating COVID-19 public health messaging in Italy: Self-reported compliance and growing mental health concerns. <https://bit.ly/2UfpoW3>.

Boruchowicz, Cynthia, Florencia López Bóo, Flora Finamor Pfeifer, Guilherme A. Russo, and Tainá Souza Pacheco. 2020. Are behaviorally informed text messages effective in promoting compliance with COVID-19 preventive measures? Evidence from an RCT in the city of São Paulo. Technical Note 2021. Washington, DC: Inter-American Development Bank.

Bottan, Nicolas, Bridget Hoffmann, and Diego Vera-Cossio. 2020. The unequal impact of the coronavirus pandemic: Evidence from seventeen developing countries. *PLoS ONE* 15(10): e0239797.

Carfora, Valentina and Patrizia Catellani, 2021. The effect of persuasive messages in promoting home-based physical activity during COVID-19 pandemic. *Frontiers in Psychology* 12L doi: 10.3389/fpsyg.2021.644050.

Falco, Paolo, and Sarah Zaccagni. 2021. Promoting social distancing in a pandemic: Beyond the good intentions. *PLoS ONE* 16(12): e0260457.

Favero, Nathan and Morgens Jin Pedersen. 2020. How to encourage ‘‘togetherness by keeping apart’’ amid COVID-19? The ineffectiveness of prosocial and empathy appeals. *Journal of Behavioral Public Administration* 3 (2).

Garcia-Subirats, Irene, Ingrid Vargas, Amparo Susana Mogollón-Pérez, Pierre De Paepe, Maria Rejane da Silva, Jean Pierre Unger, Carme Borrell, and Maria Luisa Vázquez. 2014. Inequities in access to health care in different health systems: A study in municipalities of central Colombia and north-eastern Brazil. *International Journal for Equity in Health* 13(10). <https://doi.org/10.1186/1475-9276-13-10>.

Hacquin, Anne-Sophie, Hugo Mercier, Coralie Chevallie. 2020. Improving preventive health behaviors in the COVID-crisis: A messaging intervention in a large nationally representative sample. PsyArXiv Preprints [10.31234/osf.io/nyvmg](https://doi.org/10.31234/osf.io/nyvmg)

Hume, Susannah, Peter John, Michael Sanders, and Emma Stockdale. 2021. Nudge in the time of coronavirus: The compliance to behavioral messages during crisis. *Journal of Behavioral Public Administration* 4(2): <https://doi.org/10.30636/jbpa.42.238>

Jordan, Jillian, Erez Yoeli, and David G. Rand. 2021. Don’t get it or don’t spread it? Comparing self-interested versus prosocially framed COVID-19 prevention messaging. *Nature Scientific Reports* 11: 20222.

Lunn, Peter, Cameron Belton, Ciarán Lavin, Féidhlim McGowan, Shane Timmons, and Dierdre Robertson. 2020b. Using behavioural science to help fight the coronavirus. *Journal of Behavioral Public Administration* 3(1). <https://doi.org/10.30636/jbpa.31.147>.

Sanders, Michael, Emma Stockdale, Susannah Hume, and Peter John. 2020. Loss aversion fails to replicate in the coronavirus pandemic: Evidence from an online experiment. *Economics Letters*. <https://doi.org/10.1016/j.econlet.2020.109433>.

Sasaki, Shusaku, Hirofumi Kurokawa, and Fumio Ohtake. 2021. Effective but fragile? Responses to repeated nudge-based messages for preventing the spread of COVID-19 infection. *The Japanese Economic Review* 72: 371–408.

Utych, Stephen M., and Luke Fowler. 2020. Age-based messaging strategies for communication about COVID-19. *Journal of Behavioral Public Administration* 3(1). <https://doi.org/10.30636/jbpa.31.151>.

Table S1. Testing for attrition bias: probit regression results; dependent variable is probability that baseline participant was dropped from sample because of (i) implementation

issue (n = 80), (ii) incomplete observation (n = 53),

or (iii) either (n = 109); marginal effects (s.e.)

| **Variable** | (i) Implementation issue | (ii) Incomplete | (ii) Either (attrition) |
| --- | --- | --- | --- |
| *private* | 0.028 | -0.005 | 0.031 |
|  | (0.073) | (0.016) | (0.073) |
| *public* | -0.031 | 0.022 | -0.01 |
|  | (0.039) | (0.014) | (0.035) |
| *combined* | -0.033 | -0.003 | -0.031 |
|  | (0.039) | (0.017) | (0.037) |
|  |  |  |  |
| Observations | 1330 | 1330 | 1330 |
| Chi-squared | 2.143 | 5.952 | 2.350 |

Standard errors are clustered at baseline survey session-level.

*** p<0.01, ** p<0.05, * p<0.1.

Table S2. Covariate balance: ordinary least squares regression results; dependent

variable is probability of assignment to treatment; coefficient (s.e.)

| **Variable** | ***private*** | ***public*** | ***combined*** |
| --- | --- | --- | --- |
| *older* | -0.015 | -0.033 | -0.041 |
|  | (0.051) | (0.05) | (0.049) |
| *female* | -0.021 | 0.038 | -0.009 |
|  | (0.047) | (0.047) | (0.047) |
| *poor* | -0.055 | 0.029 | -0.056 |
|  | (0.06) | (0.057) | (0.058) |
| *work* | -0.182* | -0.100 | 0.034 |
|  | (0.104) | (0.094) | (0.087) |
| *relatives in hh* | -0.006 | -0.038 | -0.129 |
|  | (0.089) | (0.089) | (0.08) |
| *no. people in hh* | 0.006 | 0.012 | 0.003 |
|  | (0.015) | (0.016) | (0.014) |
| *elder in hh* | 0.022 | 0.019 | -0.015 |
|  | (0.072) | (0.063) | (0.065) |
| *elder parent* | 0.029 | -0.012 | -0.026 |
|  | (0.078) | (0.07) | (0.072) |
| *health* | -0.051 | -0.074 | -0.118** |
|  | (0.054) | (0.052) | (0.054) |
| *comorbidity self* | 0.000 | 0.123 | 0.114 |
|  | (0.088) | (0.079) | (0.075) |
| *comorbidity parents* | -0.12** | -0.129** | -0.059 |
|  | (0.052) | (0.053) | (0.048) |
| *left wing* | -0.023 | -0.082 | -0.025 |
|  | (0.05) | (0.05) | (0.049) |
| *right wing* | 0.087 | -0.018 | 0.024 |
|  | (0.078) | (0.081) | (0.077) |
| *knows Covid19 case* | 0.004 | 0.041 | -0.046 |
|  | (0.068) | (0.067) | (0.069) |
| *knows Covid19 death* | 0.106 | 0.179 | 0.044 |
|  | (0.148) | (0.126) | (0.159) |
|  |  |  |  |
| Observations | 484 | 492 | 511 |
| R-squared | 00.068 | 00.064 | 00.044 |
| Joint signficance^a^ | 00.467 | 00.542 | 00.924 |

All regressions include (n=18) *localidad* fixed effects.

^a^Probability-value of test that all covariates are jointly significant predictors of treatment.

*** p<0.01, ** p<0.05, * p<0.1.

Table S3. Most important reason for complying and not complying

with each nonpharmaceutical intervention at baseline:

Percentage of respondents selecting each reason

| **Reason** | ***hand washing*** | ***face mask*** | ***cleaning*** | ***stay home*** | ***social distancing*** |
| --- | --- | --- | --- | --- | --- |
| *Panel A: Most important reason for complying* | | | | | |
|  | (n = 1221) | (n = 1221) | (n = 1221) | (n = 1221) | (n = 1221) |
| Want to avoid getting seriously ill | 22.0 | 32.4 | 20.8 | 28.6 | 31.9 |
| Want to avoid infecting family | 70.9 | 55.7 | 71.5 | 61.1 | 57.8 |
| Want to avoid infecting cohabitators | 6.1 | 9.3 | 3.8 | 5.9 | 8.3 |
| Want to avoid infecting friends | 0.1 | 0.1 | 0.4 | 0.4 | 0.3 |
| Want to avoid disapproval | 0.5 | 0.7 | 0.2 | 0.4 | 0.4 |
| Want to avoid government sanctions | 0.2 | 1.7 | 0.3 | 2.7 | 0.2 |
| Don’t know | 0.1 | 0.0 | 1.5 | 0.2 | 0.4 |
| Don’t want to answer | 0.2 | 0.2 | 1.4 | 0.7 | 0.7 |
| *Panel B: Most important reason for not complying** | | | | | |
|  | (n = 1023) | (n = 372) | (n = 1022) | (n = 618) | (n = 774) |
| Not convenient | 1.6 | 3.5 | 4.0 |  |  |
| Not comfortable | 1.8 | 33.3 | 7.3 |  |  |
| I forget to do it | 81.0 | 48.9 | 72.7 |  |  |
| Lack requisite supplies | 3.0 | 2.7 | 2.6 |  |  |
| Will not prevent spread Covid19 | 0.3 | 0.8 | 4.2 |  |  |
| Not concerned about spread Covid19 | 0.6 | 0.5 | 0.3 |  |  |
| Don't remember | 9.9 | 5.4 | 7.0 |  |  |
| Don't want to answer | 1.9 | 4.8 | 1.8 |  |  |
|  |  |  |  |  |  |
| Work outside home |  |  |  | 10.0 |  |
| Socialize |  |  |  | 13.6 |  |
| Study with colleagues |  |  |  | 1.3 |  |
| Participate in sports or exercise |  |  |  | 18.1 |  |
| Take a walk |  |  |  | 17.8 |  |
| Shop for items other than food |  |  |  | 21.2 |  |
| Other |  |  |  | 13.1 |  |
| Don't remember |  |  |  | 1.8 |  |
| Don't want to answer |  |  |  | 3.1 |  |
|  |  |  |  |  |  |
| Difficult in crowded locations |  |  |  |  | 42.5 |
| Job requires close contact |  |  |  |  | 3.4 |
| Sports requires close contact |  |  |  |  | 1.0 |
| Shopping requires close contact |  |  |  |  | 26.4 |
| Not convenient |  |  |  |  | 0.5 |
| I forget to do it |  |  |  |  | 16.3 |
| Will not prevent spread Covid19 |  |  |  |  | 0.3 |
| Not concerned about spread Covid19 |  |  |  |  | 0.6 |
| Other |  |  |  |  | 3.9 |
| Don't remember |  |  |  |  | 3.2 |
| Don't want to answer |  |  |  |  | 1.9 |

*Among participants who reported not always complying.

Table S4. Change in outcome means from baseline to endline, by treatment

| **Variable** | **Nobs.**  **baseline**  **(all)** | **Mean**  **baseline**  **(all)** | **Change**  **(control)** | **Change**  **(private)** | **Change**  **(public)** | **Change**  **(combined)** |
| --- | --- | --- | --- | --- | --- | --- |
| Concern |  |  |  |  |  |  |
| *likelihood infection* | 1214 | 2.67 | -0.13 | 0.03 | 0.02 | 0.00 |
| *concern self* | 1219 | 2.67 | -0.10 | 0.09 | -0.16 | -0.03 |
| *concern friends* | 1218 | 3.34 | -0.14 | 0.00 | -0.14 | -0.10 |
| *concern household* | 1208 | 3.70 | -0.05 | 0.04 | -0.04 | -0.01 |
| *concern community* | 1219 | 3.18 | -0.20 | -0.06 | -0.15 | -0.03 |
| *concern index* | 1221 | 0.00 | 0.00 | 0.17 | 0.05 | 0.11 |
| Recent compliance |  |  |  |  |  |  |
| *hand washing* | 1195 | 76.80 | 8.48 | 7.26 | 8.85 | 8.14 |
| *face mask* | 1219 | 93.57 | 2.56 | 2.61 | 1.21 | 1.19 |
| *cleaning* | 1163 | 3.90 | 0.22 | 0.04 | 0.31 | 0.33 |
| *stay home* | 1209 | 5.98 | -0.25 | -0.15 | -0.17 | -0.25 |
| *social distancing* | 1210 | 80.26 | 2.28 | 3.23 | 4.71 | 2.60 |
| *recent compliance index* | 1221 | 0.02 | -0.00 | 0.00 | 0.03 | -0.00 |
| Intentended compliance |  |  |  |  |  |  |
| *hand washing intention* | 1211 | 90.52 | 1.90 | 1.49 | 1.67 | 1.83 |
| *face mask intention* | 1212 | 94.81 | 1.77 | 2.59 | 2.30 | 0.32 |
| *cleaning intention* | 1165 | 5.07 | -0.21 | 0.03 | -0.04 | 0.02 |
| *stay home intention* | 1173 | 6.17 | -0.10 | -0.06 | -0.15 | -0.13 |
| *social dist. intention* | 1209 | 88.53 | 0.49 | -0.13 | 2.00 | 2.28 |
| *intended compliance index* | 1220 | -0.00 | 0.00 | 0.03 | 0.03 | 0.01 |

Table S5. Treatment effect heterogeneity for recent compliance;

ordinary least squares regression results

| Treatments |  |
| --- | --- |
| *private* | -0.132* |
|  | (0.0708) |
| *public* | -0.246** |
|  | (0.102) |
| *combined* | -0.159** |
|  | (0.0736) |
| *private×left wing* | 0.106 |
|  | (0.0695) |
| *public×left wing* | 0.251*** |
|  | (0.0731) |
| *combined×left wing* | 0.142** |
|  | (0.0701) |
| *private×no. people in hh* | 0.0164 |
|  | (0.0200) |
| *public×no. people in hh* | 0.0478** |
|  | (0.0224) |
| *combined×no. people in hh* | 0.0248 |
|  | (0.0222) |
| *private×poor* | 0.140* |
|  | (0.0764) |
| *public×poor* | 0.117 |
|  | (0.109) |
| *combined×poor* | 0.0882 |
|  | (0.0771) |
|  |  |
| Observations | 1079 |
| R-squared | 0.456 |

The dependent variable is the endline *recent compliance index*. Independent variables are the baseline *recent compliance, index*, *older, female, poor, work, relatives in hh, no. people in hh, elder in hh, elder parent, poor health, comorbidity self, comorbidity parents, left wing, right wing, knows Covid19 case*, *knows* C*ovid19 death,* and (n=18) *localidad* fixed effects. Standard errors are clustered at baseline survey session-level.

*** p<0.01, ** p<0.05, * p<0.1.
